# A phase 1 study of dimdazenil to evaluate the pharmacokinetics, food effect and safety in Chinese healthy subjects

**DOI:** 10.3389/fphar.2023.1226014

**Published:** 2023-08-01

**Authors:** Fei Wang, Jingjing He, Yanling Zhou, Lijun Ye, Bei Li, Zhiyuan Ma, Chunyan Chen, Ruoxi Zhang, Zhaocun Lin, Jinshan Tang, Zhiping Jin, Yu Jiang, Nengming Lin

**Affiliations:** ^1^ Phase 1 Clinical Trial Center, Affiliated Hangzhou First People’s Hospital, Zhejiang University School of Medicine, Hangzhou, China; ^2^ Key Laboratory of Clinical Cancer Pharmacology and Toxicology Research of Zhejiang Province, Affiliated Hangzhou First People’s Hospital, Zhejiang University School of Medicine, Hangzhou, China; ^3^ Shanghai Research Institute, Zhejiang Jingxin Pharmaceutical Co., Ltd., Shanghai, China; ^4^ Zhejiang Jingxin Pharmaceutical Co., Ltd., Shaoxing, China; ^5^ West lake Laboratory of Life Sciences and Biomedicine of Zhejiang Province, Hangzhou, China; ^6^ Cancer Center, Zhejiang University, Hangzhou, China

**Keywords:** insomnia, dimdazenil, GABAA receptor, partial positive allosteric agonist, pharmacokinetics, safety

## Abstract

**Background and objective:** As a partial positive allosteric modulator of the gamma-aminobutyric acid A (GABAA) receptor, dimdazenil was used for the treatment of insomnia with the potential to alleviate associated side effects compared to full agonists. The objective of this trial is to assess the safety, tolerability, food effect and pharmacokinetics following single and multiple doses of dimdazenil in Chinese healthy subjects.

**Methods:** In this phase 1 trial, 36 healthy subjects aged ≥18 years were assigned to receive a single dose of 1.5, 2.5, or 5 mg dimdazenil, with each dose cohort consisting of 12 subjects, and 14 subjects were assigned to receive a multiple 2.5 mg daily dose of dimdazenil for 5 days. Safety, tolerability, and pharmacokinetic characteristics were evaluated.

**Results:** Of the 50 subjects enrolled and 49 completed the trial, the incidences of treatment-emergent adverse events (AEs) in the single-dose groups of 1.5, 2.5, and 5 mg were 16.7%, 58.3% and 66.7% respectively, while 61.5% in the multiple-dose group. There were no serious AEs, deaths, AEs leading to discontinuation or AEs of requiring clinical intervention in any treatment groups. The most treatment-emergent AEs were dizziness (*n* = 4, 8.2%), hyperuricemia (*n* = 2, 6.1%), upper respiratory tract infection (*n* = 2, 6.1%), diastolic blood pressure decreased (*n* = 2, 6.1%), blood TG increased (*n* = 2, 6.1%) and RBC urine positive (*n* = 2, 6.1%). All AEs were mild-to-moderate and transient, and no severe AEs were documented in any study phase. The PK profile of dimdazenil and its active metabolite Ro46-1927 was linear across 1.5–5 mg oral doses in humans. The median T_max_ for dimdazenil was in the range of 0.5–1.5 h, and the apparent terminal t_1/2z_ ranged from 3.50 to 4.32 h. Taking Dimdazenil with food may delay T_max_ and decrease C_max_, without affecting the total exposure (AUC). No relevant accumulations of dimdazenil and Ro 46–1927 were observed in multiple-dose group.

**Conclusion:** Dimdazenil was generally well tolerated in healthy Chinese subjects after single and 5 days-multiple dosing. The pharmacokinetic properties of dimdazenil are compatible with a drug for the treatment of insomnia.

**Clinical Trial Registration**: chinadrugtrials.org.cn, identifier CTR20201978

## 1 Introduction

As a common ailment among adults, insomnia affects up to 30% of people worldwide (Cunnington et al., 2013; Hertenstein et al., 2022; Perlis et al., 2022). About 10% of the general population has suffered symptoms of distress or day-time functional impairment caused by insomnia (Bhaskar et al., 2016). Chronic insomnia imposes substantial economic burdens on society, significantly affecting both personal and professional lives while also increasing the risk of various health issues (Riemann et al., 2015; Kay-Stacey and Attarian, 2016). Severe insomnia can diminish work efficiency, impair vigilance level, and even lead to malignant accidents and huge losses. Moreover, numerous medical and psychiatric conditions can disrupt sleep patterns, particularly anxiety, depression, and other mental health issues (Buysse, 2013; Williams Buckley et al., 2020). Cognitive behavioral therapy for insomnia (CBT-I) is widely recommended as a primary treatment option treatment in many clinical guidelines due to its lasting effectiveness and minimal side effects (Riemann et al., 2017; Alimoradi et al., 2022). However, the limited accessibility of CBT-I poses challenges to its real-world implementation (Wilson et al., 2019). As a supplement or combination of behavioral cognitive therapy, pharmacologic treatments for chronic insomnia remain common (Wilson et al., 2019).

Current pharmacotherapies include gamma-aminobutyric acid A (GABAA) receptor positive allosteric modulators such as benzodiazepines (BZDs) or non-benzodiazepine drugs (the so-called Z-drugs, zolpidem, zopiclone, and zaleplon, *etc.*), dual orexin receptor antagonists (DORAs), sedative antidepressants and melatonin receptor agonists (Qaseem et al., 2016; Sutton, 2021). BZDs have been associated with residual sedation, memory loss, and addiction, which may limit their clinical application (Griffin et al., 2013). Although Z-drugs are considered safer than traditional BDZs, they still produce occasional dependence, tolerance, and confusional arousals in at-risk populations (Brandt and Leong, 2017; Atkin et al., 2018). Additionally, it is important to acknowledge that both BZDs and Z-drugs are also associated with a significant risk of falls and fractures among the elderly (Patel et al., 2018; Samara et al., 2020). Orexin are key neurotransmitters of arousal in the central nervous system (Roecker et al., 2016), and many studies have demonstrated that regulating the orexin system is a potential therapy for insomnia or other sleep disorders (Muehlan et al., 2020; Sun et al., 2021). DORAs such as suvorexant, lemborexant, and daridorexant have shown effective in treating insomnia, with fewer influences on daytime performance and no rebound insomnia after drug withdrawal (Kuriyama and Tabata, 2017; Scott, 2020; Markham, 2022). However, their probability of worsening depression and inducing complex sleep behaviors has been well-documented (Earl and Van Tyle, 2020). Exogenous melatonin and melatonin agonists can promote sleep and regulating circadian rhythms (Wilson et al., 2019). The represented melatonin agonist ramelteon has demonstrated efficacy in the management of insomnia and been approved for treating insomnia (Reynoldson et al., 2008). Despite being non-potential for abuse or dependence, melatonin agonists may induce elevated liver enzymes, upper respiratory tract infections, and urinary tract infections, even influence reproductive function in women (Richardson and Wang-Weigand, 2009; Fernando and Rombauts, 2014; Perlis et al., 2022). In addition, the utilization of sedative antidepressants, such as trazodone, doxepin and lower doses of mirtazapine, may be considered, when comorbid depression is present or in situations where other treatment modalities have been ineffective (Perlis et al., 2022). Howerer, evidence for their efficacy in insomnia when used alone is relatively weak (Hajak et al., 2001; Winokur et al., 2003; Mendelson, 2005). Therefore, there is an unmet need to develop better pharmacologic treatments for insomnia.

Structurally belonging to the class of BZD drugs, dimdazenil is a novel partial positive allosteric modulator of the GABAA receptor for the treatment of insomnia (Zhang et al., 2018). Animal studies have provided evidence that long-term use of full agonists of GABAA receptor can lead to adaptive changes in the receptors, diminishing the effectiveness of the endogenous neurotransmitter GABAA and resulting in withdrawal symptoms (Bateson, 2002; Wilson et al., 2019). Dimdazenil, being a partial agonist, produces a lower maximal potentiation of GABAA receptors than a full agonist (Walsh et al., 2009). This characteristic may contribute to a lower potential for undesired effects while maintaining desired therapeutic effects. *In vitro* studies have shown that dimdazenil exhibits highers selectivity for the alpha-1 subtype of GABAA receptors compared to other subtypes. The agonistic potency of dimdazenil in the alpha-1 subtype is approximately three times than that in the alpha-5 subtype (with EC_50_ value of 18 ± 6 nM and 53 ± 6 nM, respectively). Highly expressed in the cortex, the alpha-1 subtype probably mediates the sedative and hypnotic effects (Munro et al., 2009; Luppi et al., 2017; Sente et al., 2022). The high selectivity of dimdazenil to alpha-1 subtype may also decrease undesirable effects mediated by acting other subtypes (Sigel and Steinmann, 2012).

Prior studies of dimdazenil in elderly and non-elderly adult primary insomnia patients have demonstrated positive results at doses of 1.5 and 2.5 mg (Walsh et al., 2009; Walsh et al., 2010). Given the potential clinical benefits and its properties as a partial agonist, a phase 1 study was conducted on Chinese healthy subjects to assess the pharmacokinetics (PK) and safety of the single-dose and multiple-dose administration of dimdazenil, as well as evaluate the potential effect of food on the disposition of dimdazenil.

## 2 Methods

### 2.1 Study design and oversight

This was a 2-part, non-randomized, single-center, open-label, phase 1 study to evaluate the safety/tolerability, PK, and potential effect of food on orally administered dimdazenil in Chinese healthy subjects.

Single-dose part (Part 1) included two phases, the fasted phase was followed by the fed phase. After a screening period, subjects of three dose groups (1.5, 2.5, and 5 mg) were treated with a single oral dose of dimdazenil capsule in the morning while in the fasted state. After the fasted phase study completion, participants in 2.5 mg dose group were administered 2.5 mg dimdazenil underfed after a washout period of 14 days. During this phase, a high-fat meal was provided in the morning, and dimdazenil capsules were administered 30 min after the beginning of the meal to investigate the effect of food on PK. Following drug administration, each PK sample was taken on Day 1 at the following time points: pre-dose, 15 min, 30 min, 1, 1.5, 2, 2.5, 3, 3.5, 4, 5, 6, 8, 12 h post-dose, and at 24, 36, 48 and 72 h post-dose.

In the multiple-dose part (Part 2), subjects received a daily 2.5 mg dose of dimdazenil for 5 days in the evening (about 21:00). Plasma samples were collected on Day 1 at the following time points: 15 min, 30 min, 1, 1.5, 2, 2.5, 3, 3.5, 4, 5, 6, 8, and 12 h post-dose, and pre-dose in the evening of Day 2, 3, 4 and 5. After the last drug administration, PK samples were taken at 15 min, 30 min, 1, 1.5, 2, 2.5, 3, 3.5, 4, 5, 6, 8, 12, 24, 36, 48, and 72 h.

### 2.2 Participants

A total of 50 healthy subjects included in this trial, 36 subjects were in Part 1 and 14 subjects were in Part 2. Eligible participants were aged ≥18 years with a body mass index (BMI) of 19–26 kg/m^2^ and a body weight of ≥50 kg (men) or 45 kg (female). Subjects were generally healthy at screening and/or prior to the initial administration of a trial drug, the intraocular pressure of both eyes was in the range of 10–21 mmHg, and the oxygen saturation of finger pulse blood was within the scope of 95%–100%. Concomitant medications were not permitted during the study, guaranteeing that the investigation of safety/tolerability and PK variables would not be affected by other drugs.

The main exclusion criteria were a history of significant medical, neurologic, or serious psychiatric illness. Besides, any caffeinated, alcoholic xanthine-rich food or drink consumed within 48 h before dosing was not allowed.

The study was conducted at Affiliated Hangzhou First People’s Hospital, Zhejiang University School of Medicine. The protocol was approved by the Center for Drug Evaluation, NMPA. Regulatory and institutional review board or independent ethics committee approval was obtained for each subject and written informed consent was obtained from all participants in accordance with local regulations.

### 2.3 Evaluation

The objective of this study was to evaluate the safety, tolerability and PK characteristics of single- and multiple-dose oral administration of dimdazenil in healthy adults, as well as the effects of food on the PK of dimdazenil. In this study, participants were monitored as inpatients, with day −1 considered to be the day before initial administration. Participants were administered treatment on day 1, and assessed for safety/tolerance and PK characteristics at selected times during this study. Participants were considered to complete the trial according to the protocol after their final planned safety follow-up visit.

Venous blood samples were taken pre-dose and at specified time points after drug administration. Evaluated single-dose plasma PK parameters for dimdazenil included maximum observed concentration (C_max_), time to reach the maximum observed concentration (T_max_), apparent terminal elimination half-life (t_1/2z_), an area under the concentration–time curve (AUC) from time of administration to 24 h (AUC_0–24h_), AUC from time of administration to infinity (AUC_0-∞_), AUC from time of administration to the last measurable concentration (AUC_0-t_), apparent clearance (CL_z_), and apparent volume of distribution (V_z_). Multiple-dose PK parameters calculated for dimdazenil included a maximum observed concentration in steady-state (C_max,ss_), AUC during the dosing interval (AUC_0-τ_), accumulation ratio based on C_max_ (Ra_(Cmax)_) and AUC_0-τ_ (Ra_(AUC)_),AUC_0-∞,ss_, AUC_0-t,ss_, t_1/2z,_ T_max_, CL_z_ and V_z_. PK parameters were also evaluated for Ro46-1927 and Ro18-5,528, the circulating metabolites of dimdazenil.

The safety evaluation included adverse-events monitoring, vital signs (blood pressure, pulse rate and body temperature), physical examination (head and neck, skin and mucous membranes, abdomen, chest, lymph nodes, musculoskeletal, nervous system and others), intraocular pressure test, clinical laboratory tests (hematologic, biochemical, urinalysis, coagulation, urine sediment tests), and 12-lead electrocardiography (ECG). Treatment-emergent adverse events (TEAEs) were recorded and analyzed based on their frequency, nature and intensity. Adverse events (AEs) were counted once per subject by coded preferred term, considering the worst severity and strongest causality. The severity grading utilized the Common Terminology Criteria for Adverse Events (CTCAE), version 5.0, and coding was performed with Medical Dictionary for Regulatory Activities (MedDRA), version 23.0. All reported AEs were assessed in terms of the occurrence time, the severity level, the duration, the measures taken, the ultimate outcome, and the prognosis.

### 2.4 Statistical analyses

The participants who received at least one dose of dimdazenil were all included in the safety analysis. And the participants who received all planned doses of the regimen with valid post-drug concentration data were all included in the PK analysis.

Plasma PK parameters were analyzed by WinNonlin software, version 8.1 or higher (Certara Institute), other analyses were performed using SAS software, versions 9.4 and higher (SAS Institute). The PK analysis utilized the typical non-compartmental approach, and the PK parameters were descriptively summarized by doses, including the number of observations, arithmetic mean, geometric mean, standard deviation (SD), coefficient of variation (CV), 95% confidence interval (CI) of the mean, minimum, quartile, median, maximum and quartile, as appropriate. Descriptive statistics were used to summarize demographic and baseline characteristics, including frequencies, percentages, means, SD, medians, quartiles, minimum values, and maximum values. Safety and tolerability results were analyzed descriptively by treatment cohorts.

## 3 Results

### 3.1 Baseline characteristics and subject disposition

**FIGURE 1:**
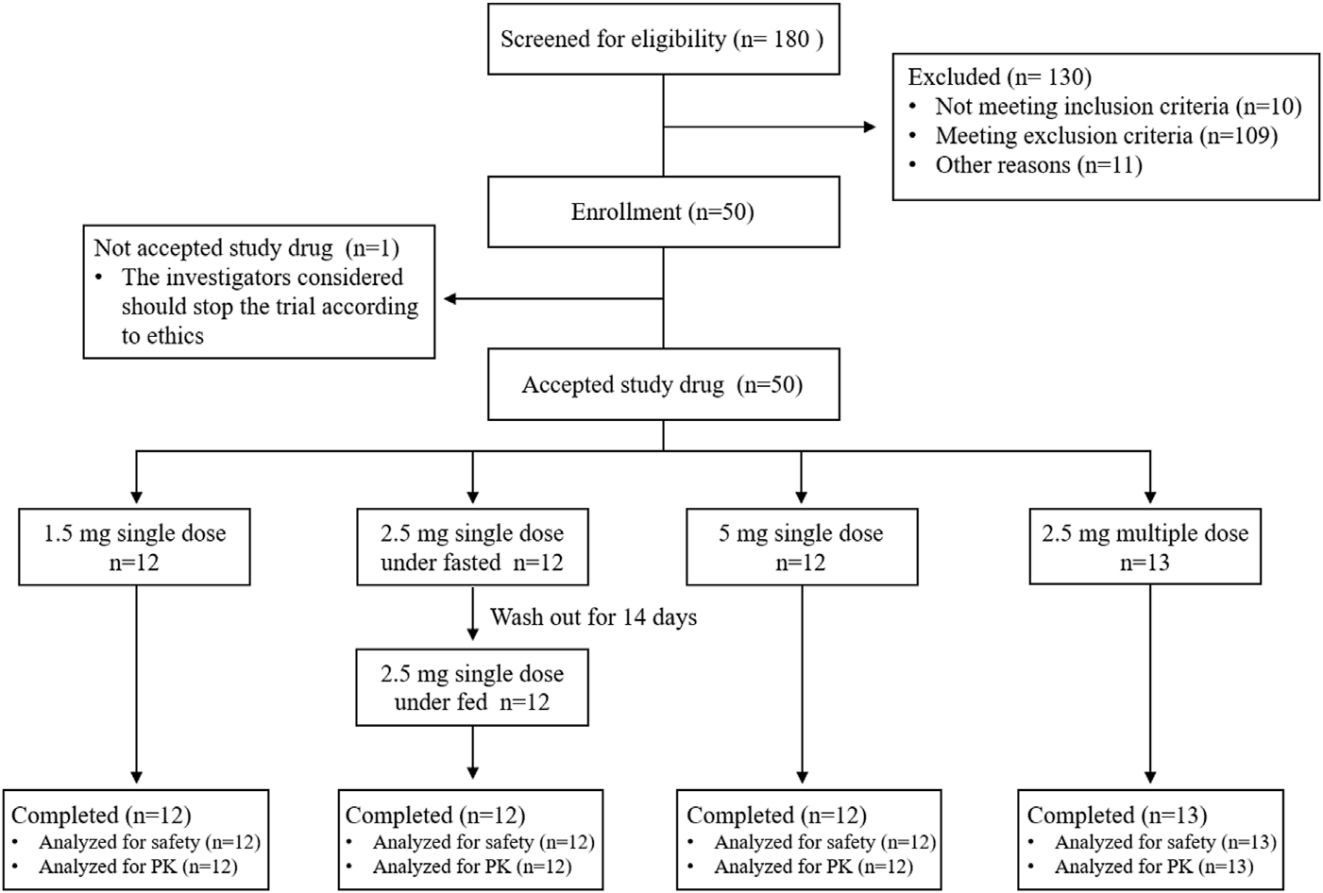
Schematic flow diagram of this study.

From 20 October 2020, to 14 December 2020, 50 healthy subjects were enrolled in the study, as shown in the trial flowchart in Figure 1. One participant in the multiple-dose part did not receive dimdazenil capsules and was excluded from the PK and safety analyses. 49 subjects accepted drug administration according to protocol and completed all the experimental procedures. There were no concomitant medications or non-drug therapies for any participants, and no serious deviation of the protocol in this study. PK parameters were calculated according to the actual sampling time.

The characteristics of the participants at baseline according to the assigned groups are shown in Table 1. Overall, the mean age was 28.4 years (range 20–50 years), the mean height was 166.46 cm (146.5–179 cm), the mean weight was 62.16 kg (46.4–77.6 kg), and the mean BMI was 22.38 kg/m^2^ (19.4–25.9 kg/m^2^). Demographic and baseline characteristics were similar between treatment groups.

**TABLE 1:** Demographic and clinical characteristics of the participants at baseline^a^.

Characteristic	Single dose			Multiple-dose	Total
	1.5 mg	2.5 mg	5 mg	2.5 mg	
N	12	12	12	13	49
Age, yr	28.3 (20–40)	26.7 (21–35)	28.8 (21–39)	29.8 (20–50)	28.4 (20–50)
Sex, no. (%)					
Men	7 (58.3)	10 (83.3)	9 (75.0)	9 (69.2)	35 (71.4)
Female	5 (41.7)	2 (16.7)	3 (25.0)	4 (30.8)	14 (28.6)
Height, cm	166.25 (154–176)	168.46 (158.5–179)	165.13 (146.5–177.5)	166.04 (149–177)	166.46 (146.5–179)
Weight, kg	60.32 (50.5–74.3)	62.74 (56.9–72.6)	62.34 (46.4–71.4)	63.15 (47.7–77.6)	62.16 (46.4–77.6)
BMI, kg/m^2^	21.78 (20.5–25)	22.17 (19.4–25.2)	22.74 (20.7–25.7)	22.78 (19.7–25.9)	22.38 (19.4–25.9)

^a^ Age, height, weight and BMI, data are presented as means (range). One participant in the multiple-dose part did not receive the study drug regimen according to the protocol and was excluded from the PK, and safety analyses.

### 3.2 Safety

All reported TEAEs in this study are presented in Table 2. There were no serious AEs, deaths, AEs leading to discontinuation or AEs requiring clinical intervention in any treatment groups. All clinical AEs were mild-to-moderate and transient, no severe AEs were documented. In total, 39 AEs occurred in 25 (51.0%) subjects during all study cohorts, 29 AEs occurred in 18 (36.7%) subjects were considered related to the trial drug, and the remaining 10 AEs were considered unrelated to study treatment. 37 of the reported AEs were considered as mild, and 2 as moderate. The incidences of TEAEs in the single dose of 1.5, 2.5, and 5 mg groups were 16.7%, 58.3%, and 66.7% respectively, and 61.5% in the 2.5 mg multiple dose group. For single dose administration, the incidence of TEAEs was significantly lower at the 1.5 mg dose compared to the 5 mg dose (*p* = 0.013), while there was no significant difference among the other single dose groups (*p* < 0.05).

**TABLE 2:** Summary of treatment-emergent adverse events.

	Single dose			Multiple-dose	Total
	1.5 mg (N = 12)	2.5 mg^a^ (N = 12)	5 mg (N = 12)	2.5 mg (N = 12)	N = 49
Number of subjects (percent)					
Any AEs	2 (16.7)	7 (58.3)	8 (66.7)	8 (61.5)	25 (51.0)
Dizziness	0 (0)	0 (0)	4 (33.3)	0 (0)	4 (8.2)
hyperuricemia	0 (0)	1 (8.3)	2 (16.7)	0 (0)	3 (6.1)
Upper respiratory tract infection	0 (0)	1 (8.3)	0 (0)	2 (15.4)	3 (6.1)
Myalgia	0 (0)	0 (0)	0 (0)	1 (7.7)	1 (2.0)
Nasal obstruction	0 (0)	0 (0)	0 (0)	1 (7.7)	1 (2.0)
Sinus tachycardia	0 (0)	0 (0)	0 (0)	1 (7.7)	1 (2.0)
Ocular hypertension	0 (0)	1 (8.3)	0 (0)	0 (0)	1 (2.0)
DBP decreased	0 (0)	0 (0)	1 (8.3)	2 (15.4)	3 (6.1)
ECG PR shortened	0 (0)	1 (8.3)	0 (0)	0 (0)	1 (2.0)
ECG T wave abnormal	0 (0)	0 (0)	0 (0)	1 (7.7)	1 (2.0)
Heart rate decreased	0 (0)	0 (0)	0 (0)	1 (7.7)	1 (2.0)
Heart rate increased	1 (8.3)	1 (8.3)	0 (0)	0 (0)	2 (4.1)
Blood bilirubin increased	1 (8.3)	0 (0)	0 (0)	0 (0)	1 (2.0)
Blood TG increased	0 (0)	0 (0)	2 (16.7)	1 (7.7)	3 (6.1)
CRE increased	0 (0)	2 (16.7)	0 (0)	0 (0)	2 (4.1)
ALT increased	0 (0)	1 (8.3)	0 (0)	0 (0)	1 (2.0)
RBC urine positive	0 (0)	1 (8.3)	2 (16.7)	0 (0)	3 (6.1)
WBC urine positive	0 (0)	1 (8.3)	1 (8.3)	0 (0)	2 (4.1)
MC count increased	0 (0)	0 (0)	0 (0)	1 (7.7)	1 (2.0)
Urinary sediment present	0 (0)	1 (8.3)	0 (0)	0 (0)	1 (2.0)
Protein urine present	0 (0)	0 (0)	1 (8.3)	0 (0)	1 (2.0)

DBP: diastolic blood pressure; ECG: electrocardiogram; TG: triglycerides; CRE: serum creatinine; ALT: alanine aminotransferase; RBC: red blood cells; WBC: white blood cells; MC: mononuclear cell.

^a^ AEs, were counted for both fasted and fed phase after the single dose administration of dimdazenil.

The most frequent TEAEs were dizziness (8.2%), hyperuricemia (6.1%), upper respiratory tract infection (6.1%), DBP decreased (6.1%), blood TG increased (6.1%) and RBC urine positive (6.1%). All the participants who suffered events were eventually recovered/cured, in remission or stable. No evident changes in vital signs, laboratory findings, ECG values or intraocular pressure from which baseline, and none of the subjects had suicidal ideation or suicidal behavior reported during the study.

### 3.3 Pharmacokinetics

**FIGURE 2:**
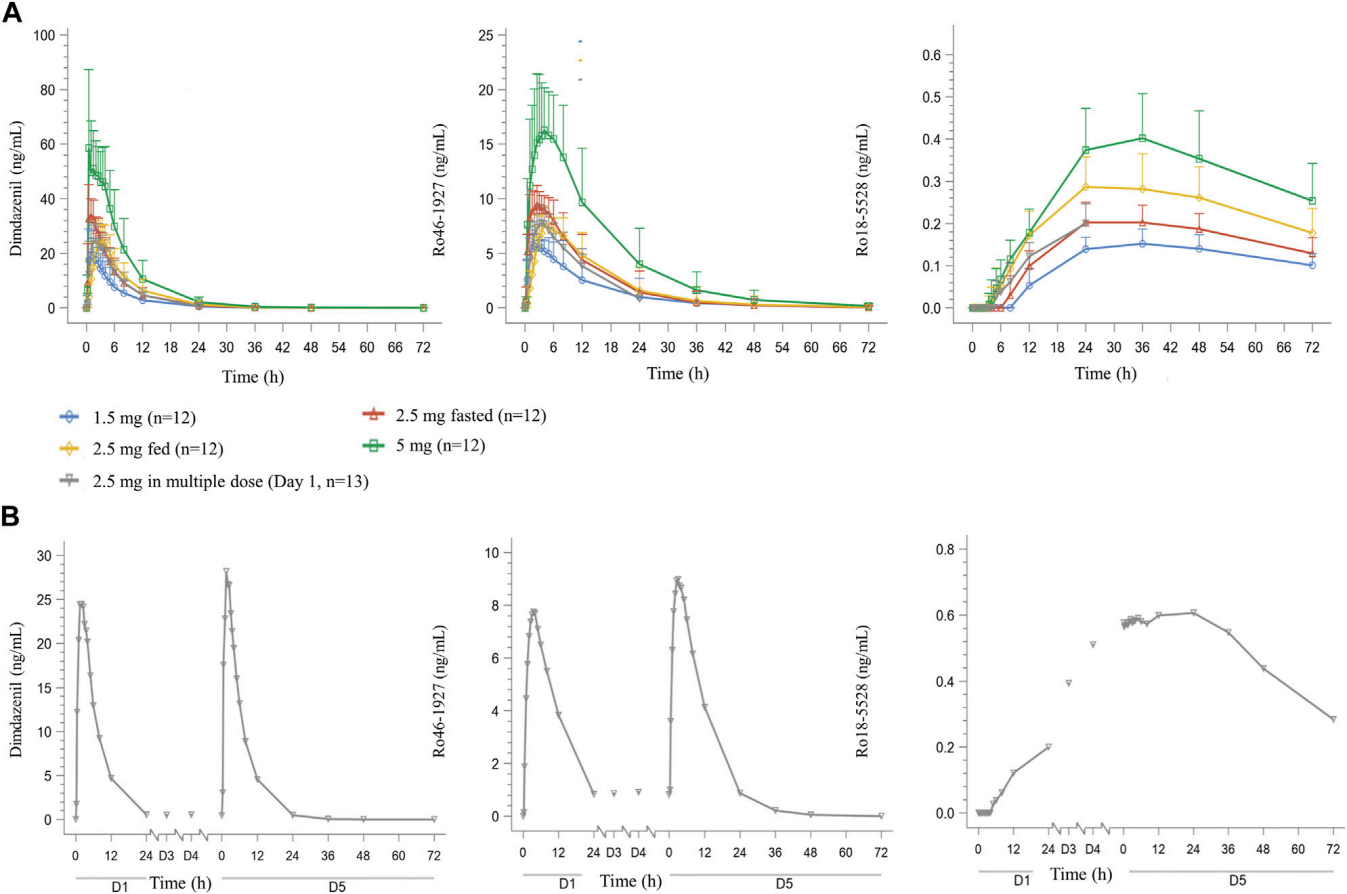
Mean (SD) plasma dimdazenil and its metabolites concentration-time curve for healthy adult subjects who received single doses of dimdazenil **(A)** and once-daily multiple doses of 2.5 mg of dimdazenil **(B)**.

The mean concentration-time profiles of dimdazenil and its circulating metabolites are depicted in Figure 2 and the PK parameters of dimdazenil are summarized in Table 3 and Table 4.

**TABLE 3:** Summary of main PK parameters of dimdazenil following single-dose administration.

Parameter	1.5 mg	2.5 mg fasted	2.5 mg fed	5 mg	Day 1 in 2.5 mg multiple dose
	N = 12	N = 12	N = 12	N = 12	N = 13
C_max_ (ng/mL)					
Mean (SD)	23.54 (6.76)	37.43 (9.30)	28.22 (6.31)	70.37 (17.37)	30.24 (6.38)
GM (CV%)	22.82 (25.4)	36.35 (26.1)	27.57 (22.8)	68.41 (25.4)	29.63 (21.2)
AUC_0-t_ (h*ng/mL)					
Mean (SD)	130.84 (86.34)	224.90 (84.34)	221.32 (91.39)	461.04 (166.65)	191.46 (52.13)
GM (CV%)	113.69 (54.8)	210.88 (39.3)	206.93 (38.8)	429.39 (43.1)	184.74 (28.7)
AUC_0-∞_ (h*ng/mL)					
Mean (SD)	132.88 (86.24)	227.05 (83.95)	224.30 (92.08)	462.44 (167.16)	194.47 (54.83)
GM (CV%)	115.99 (53.7)	213.42 (38.4)	209.97 (38.3)	430.71 (43.0)	187.21 (29.6)
AUC_0-24h_ (h*ng/mL)					
Mean (SD)	128.02 (77.55)	220.20 (75.22)	214.36 (76.04)	443.92 (151.22)	191.70 (52.32)
GM (CV%)	113.50 (50.4)	208.51 (36.1)	203.31 (34.6)	416.74 (40.4)	184.94 (28.8)
T_max_ (h)					
Median (range)	0.75 (0.5–2)	0.75 (0.5–3.5)	2.50 (1.5–5)	0.50 (0.5–5)	1.50 (0.5–3.52)
t_1/2z_ (h)					
Mean (SD)	3.80 (1.40)	3.99 (1.16)	4.14 (1.26)	4.32 (1.19)	3.50 (0.62)
V_z_ (L)					
Mean (SD)	67.93 (11.69)	65.33 (8.78)	69.04 (8.98)	71.10 (14.80)	67.69 (13.15)
CL_z_ (L/h)					
Mean (SD)	14.26 (5.70)	12.48 (4.78)	12.64 (4.48)	12.61 (5.69)	13.89 (4.08)

Data are expressed as arithmetic mean with standard deviation (SD) and geometric mean (GM) with coefficient of variation (CV), except for T_max_ which is expressed as median (range).

C_max_: maximum observed concentration; AUC: area under the concentration–time curve; AUC_0-t_: AUC, from time of administration to the last measurable concentration; AUC_0-∞_: AUC, from time of administration to infinity; AUC_0–24h_: AUC, from time of administration to 24 h; T_max_: time to reach the maximum observed concentration; t_1/2z_: apparent terminal elimination half-life; CL_z_, apparent clearance; V_z_: apparent volume of distribution.

**TABLE 4:** Summary of main PK parameter of dimdazenil following 2.5 mg multiple-dose administration.

Parameter	Statistics	2.5 mg/day (N = 13)
C_max,ss_ (ng/mL)	Mean (SD)	33.86 (7.88)
	GM (CV%)	33.02 (23.7)
AUC_0-τ_ (h*ng/mL)	Mean (SD)	198.12 (44.67)
	GM (CV%)	193.40 (23.4)
AUC_0-∞,ss_ (h*ng/mL)	Mean (SD)	201.35 (47.57)
	GM (CV%)	196.13 (24.4)
AUC_0-t,ss_ (h*ng/mL)	Mean (SD)	199.62 (47.04)
	GM (CV%)	194.49 (24.2)
T_max_ (h)	Median (range)	1.50 (0.5–2.52)
t_1/2z_ (h)	Mean (SD)	3.63 (0.69)
V_z_ (L)	Mean (SD)	67.40 (10.80)
CL_z_ (L/h)	Mean (SD)	13.25 (3.13)
R_a (AUC)_	Mean (SD)	1.05 (0.09)
	GM (CV%)	1.05 (8.4)
R_a (Cmax)_	Mean (SD)	1.13 (0.19)
	GM (CV%)	1.11 (17.8)

Data are expressed as arithmetic mean with standard deviation (SD) and geometric mean (GM) with coefficient of variation (CV), except for T_max_ which is expressed as median (range).

C_max,ss_: maximum observed concentration in steady-state; AUC: area under the concentration–time curve; AUC_0-τ_: AUC, during the dosing interval; AUC_0-∞,ss**:**_ AUC, from time of administration to the last measurable concentration in steady-state; AUC_0-t,ss_: AUC, from time of administration to the last measurable concentration in steady-state; T_max_: time to reach the maximum observed concentration; t_1/2z_: apparent terminal elimination half-life; CL_z_: apparent clearance; V_z_: apparent volume of distribution; Ra(C_max_): accumulation ratio based on C_max_, the formula is C_max,ss_ in multiple dose/C_max_ in single dose; Ra (AUC): accumulation ratio based on AUC_0-τ_, the formula is AUC_0-τ_ in multiple dose/AUC_0-t_ in single dose.

After single dose administration, dimdazenil capsules were absorbed rapidly with median T_max_ values of 0.75 (range 0.5–2), 0.75 (range 0.5–3.5), and 0.50 (range 0.5–5) h of 1.5, 2.5, and 5 mg, respectively, and the mean t_1/2z_ values were 3.80 (1.40), 3.99 (1.16), and 4.32 (1.19) h. The mean V_z_, and CL_z_ were 67.93 (11.69) L and 14.26 (5.70) L/h in 1.5 mg group, 65.33 (8.78) L and 12.48 (4.78) L/h in 2.5 mg group, 71.10 (14.80) L and 12.61 (5.69) L/h in 5 mg group, which are similar among different dose groups. A summary of the single-dose PK parameters of metabolites Ro46-1927 and Ro18-5,528 is shown in Supplementary Table S1, S2.

For dimdazenil, the estimated slope *β* (two-sided 95% CI) of C_max_, AUC_0-t_ and AUC_0-∞_ was 0.912 (0.741–1.083), 1.099 (0.802–1.396) and 1.085 (0.792–1.378), respectively. The estimated slope *β* (95% CI) of C_max_, AUC_0-t_ and AUC_0-∞_ for Ro46-1927 was 0.883 (0.762–1.005), 1.125 (0.787–1.464), and 1.123 (0.781–1.465), respectively. The estimated slope *β* (95% CI) for of C_max_, AUC_0-t_ and AUC_0-∞_ Ro18-5,528 was 0.777 (0.619–0.934), 0.817 (0.652–0.983), and 0.601 (0.208–0.994), respectively. The PK profiles of dimdazenil and Ro46-1927 were linear across 1.5–5 mg oral doses in humans, C_max_, AUC_0-t_ and AUC_0-∞_ were increased proportionally to the dose since the two-sided 95% CIs for the estimated value of *β* contained 1, while Ro18-5,528 exhibited less-than-dose-proportional increases (Supplementary Figure S1).

Over the 2.5 mg multiple dose of dimdazenil administration, a steady state was reached within 3 days with minimal accumulation, and the accumulation ratios for AUC_0-τ_ and C_max_ were 1.13 (0.19) and 1.05 (0.09), respectively. The accumulation ratios for AUC_0-τ_ and C_max_ of Ro46-1927 were 1.14 (0.13) and 1.15 (0.10), and those of Ro18-5,528 were 3.10 (0.38) and 6.05 (0.79) (Supplementary Table S3, S4). In both study parts, no trend was observed regarding the influence of sex on the PK profiles of dimdazenil and its metabolites, which are provided in Supplementary Tables S5–S7.

### 3.4 Food effect

The C_max_, AUC, and T_max_ geometric means for dimdazenil and its metabolites were compared under fed and fasted conditions to evaluate the potential impact of food. The fed/fasted geometric mean ratios (GMR) and 90% CI for C_max_, AUC_0–t_ and AUC_0-∞_ were 75.86% (64.04, 89.85), 98.13% (75.35, 127.79) and 98.39% (75.88, 127.56), respectively. The median of T_max_ values (range) were 0.75 h (0.5–3.5 h) under fasted state and 2.50 h (1.5–5 h) under fed state. Following administration of a high-fat diet, dimdazenil exhibited a 24% decreased in C_max_, a 2% decreased in AUC, and a 2 h delay in T_max_. These findings indicated that food may delay the T_max_ and decrease the C_max_, but it does not significantly affect the total exposure (AUC).

For Ro46-1927, the fed/fasted GMR for C_max_, AUC_0–t_ and AUC_0-∞_ were 81.11%, 92.18%, and 92.42%, the median T_max_ value under fed and fasted conditions were 2.50 h (range 1–8 h) and 4.00 h (range 2.5–12 h), respectively. Food consumption led to a delay in T_max_ for Ro46-1927, with a 19% decreased in C_max_ and an 8% decreased in AUC. Regarding Ro18-5,528, the fed/fasted GMR for C_max_ and AUC_0–t_ was 138.39% and 141.58%, the median of T_max_ values under fed and fasted conditions were 30.00 h (range 24–48 h) and 36.00 h (range 24–48 h). After the diet, there was a delay in Ro18-5,528 T_max_, along with an approximately 38% increase in C_max_ and a 42% increase in AUC_0-t_.

## 4 Discussion

The study provided important initial clinical pharmacology information about the PK and safety of dimdazenil in Chinese healthy subjects. The effects of food on a single dose of dimdazenil and metabolites were also studied in the trial.

After single-dose administrations, C_max_ and AUC values for dimdazenil increased with the dose, with the lowest values at 1.5 mg dose and the highest ones at 5 mg dose. The PK profiles of dimdazenil following single-dose administrations in the fasted state were characterized by absorption with a median T_max_ ranging from 0.5–1.5 h, and a mean t_1/2z_ ranging from 3.50–4.32 h across the dose groups. The elimination profiles suggested that dimdazenil could be sufficient to sustain sleep longer than recommended doses of short-acting Benzodiazepine receptor agonists (BzRAs) whose mean t_1/2_ were less than 3 h, but have a lower likelihood to induce residual effects than intermediate or long-acting BzRAs whose mean t_1/2_ were longer than 6 h (Sateia et al., 2017; Wilson et al., 2019).

Dimdazenil can be converted into an active metabolite Ro 46–1927 in humans, which exhibits similar receptor binding properties to the parent compound (Zhang et al., 2018). An additional pharmacologically inactive metabolite, Ro 18–5,528, is also found in human plasma after oral dosing, but at much lower concentrations. Following daily multiple-dose administration, no relevant accumulation of dimdazenil and Ro 46–1927 was observed, and the PK parameters were similar to those in the single-dose part, which may produce less residual sedation in next daytime. Ro 18–5,528 exhibited moderately higher concentrations on Day 5 than on Day 1 after repeat dosing. It was noteworthy that Ro 46–1927 appeared in plasma immediately after dosing on Day 1, whereas Ro 18–5,528 displayed a delayed onset of approximately 8 h following 2.5 mg *versus* single-dose administration. Furthermore, after 4 days of continuous administration, Ro 18–5,528 did not reach a steady state in plasma (Figure 2). In both study parts, no trend was observed regarding the influence of sex on the PK profiles of dimdazenil and its metabolites. Furthermore, additional analysis was conducted for the time that dimdazenil plasma concentration was above 20% of the C_max_, the result showed that the time of drug concentration reached 20% of the C_max_ was about 25–45 min, and the duration of drug concentration above 20% of the C_max_ was about 9 h (Supplementary Table S8). This may contribute to predicting the onset and maintenance time of drugs.

According to the results of food and drug interaction study, it appeared that food had a moderate effect on the rate of absorption of dimdazenil. Under the fed conditions, the mean C_max_ was lower (by about 24%) and was reached later (delayed T_max_ by about 2.5 h) compared to the fasted conditions.These results suggest that, in order to achieve faster sleep onset, dimdazenil should not be administered with or immediately after a meal.

In terms of safety and tolerability, dimdazenil was generally well tolerated, AE occurrence appeared to be dose-dependent. There were no serious AEs or deaths, and no participants dropped out from the study prematurely due to the AEs. The majority of AEs reported were mild and resolved spontaneously, which recovered in all instances. Moreover, comprehensive safety assessments including vital signs, laboratory findings, ECG values or intraocular pressure did not reveal any significant changes compared to baseline measurements. Additionally, no instances of suicidal ideation, suicidal behavior or other mental health problem were reported during the study, suggesting that dimdazenil has a relatively low impact on psychiatric risks.

The commonest AE across all groups was dizziness, reported on 8 occasions, 4 reported in 5 mg single-dose group and 4 reported in 2.5 mg multiple-dose group. Other central nervous system AEs related to benzodiazepine, including balance disorder, somnolence, headache and insomnia were not reported in this study. One subject occurred ocular hypertension (with the severity of grade I) in 2.5 mg single-dose cohort under the fed phase, which was eventually recovered without any processing, and further considered extraneous to dimdazenil by the investigator.

Nevertheless, some limitations of the study should be considered carefully. First, the participants in this trial were healthy and aged ≥18 years, which may limit the safety and tolerance findings and PK analysis in elders and insomniacs. Second, another factor that may limit the interpretation of the results is that the proportionality for the metabolites has not been confirmed, which may affect the estimation of the correlation between AEs and metabolites. In addition, the efficacy and drug dependence of dimdazenil will be further investigated in phase 2 and phase 3 clinical trials.

This phase 1 study exhibited that dimdazenil was generally well tolerated in healthy Chinese subjects at a single dose of 1.5–5 mg and multiple doses of 2.5 mg. This may be attributed to the partial GABAA agonist nature of dimdazenil, which theoretically lower the probability of some untoward effects relative to full agonists. The PK characteristics and safety findings in this study may imply that in the dose range of 1.5–5 mg, dimdazenil produce an effective function of sleep regulation for disorders in sleep initiation and sleep maintenance, with a low risk of side effects and residual sedation.

## Data Availability

The original contributions presented in the study are included in the article/Supplementary Material, further inquiries can be directed to the corresponding authors.
