# Supplementary material for: A phase 1 study of dimdazenil to evaluate the pharmacokinetics, food effect and safety in Chinese healthy subjects
Source: Front Pharmacol. 2023 Aug 1;14:1226014. doi: 10.3389/fphar.2023.1226014 (PMC10432719; doi:10.3389/fphar.2023.1226014)
Supplement: Supplementary file 1 [file DataSheet1.pdf]

Supplementary Appendix

A Phase 1 study of dimdazenil to evaluate the pharmacokinetics, food effect and safety in Chinese healthy subjects

Fei Wang[1,2†], Jingjing He[3†], Yanling Zhou[3], Lijun Ye[1], Bei Li[1], Zhiyuan Ma[1], Chunyan Chen[3], Ruoxi Zhang[3], Zhaocun Lin[3], Jinshan Tang[3], Zhiping Jin[4], Yu Jiang[3*], Nengming Lin[1,2,5,6*]

[1] *Phase 1 Clinical Trial Center,* Affiliated Hangzhou First People's Hospital, Zhejiang University School of Medicine, Hangzhou, 310006, China

[2] Key Laboratory of Clinical Cancer Pharmacology and Toxicology Research of Zhejiang Province, Affiliated Hangzhou First People's Hospital, Zhejiang University School of Medicine, Hangzhou, 310006, China

[3] Shanghai Research Institute, ZHEJIANG JINGXIN PHARMACEUTICAL CO., LTD. Shanghai, 201210, China

[4] ZHEJIANG JINGXIN PHARMACEUTICAL CO., LTD. China

[5] West lake Laboratory of Life Sciences and Biomedicine of Zhejiang Province, Hangzhou, 310024, China

[6] Cancer Center, Zhejiang University, Hangzhou, 310058, China

[†] These authors have contributed equally to this work and share first authorship.

* Correspondence: **Yu Jiang**, E-mail: yu.jiang@jingxinpharm.com and **Nengming Lin**, E-mail: lnm1013@zju.edu.cn

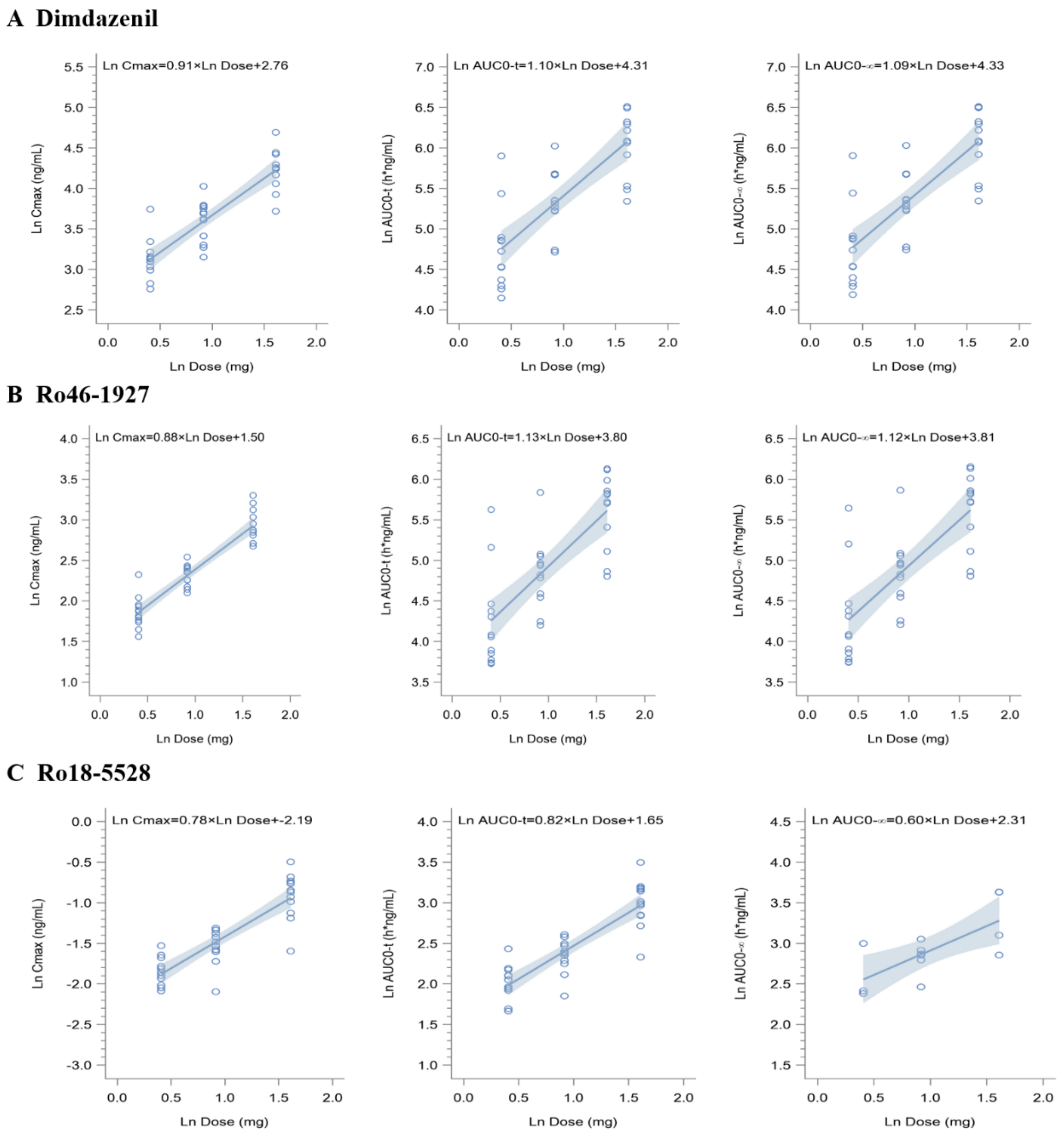

Supplementary Figure 1. The relationship between PK parameters of dimdazenil and its metabolite and dose in healthy subjects after single oral administration of dimdazenil capsules on fasting.

Supplementary Table 1. Summary of main PK parameters of Ro46-1927 following single-dose dimdazenil administration.

**Parameter**	1.5 mg N=12	2.5 mg fasted N=12	2.5 mg fed N=12	5 mg N=12	Day 1 in 2.5 mg multiple dose N=13
**C_{max}(ng/mL)**					
Mean±SD	6.55±1.40	10.22±1.33	8.29±1.06	18.99±4.04	8.51±1.68
GM	6.43	10.14	8.22	18.63	8.37
GM-CV%	19.8	13.2	12.7	20.5	19.3
**AUC_{0-t}(h*ng/mL)**					
Mean±SD	85.99±70.45	137.85±71.54	128.73±72.89	297.69±115.88	94.41±20.98
GM	70.41	125.56	115.75	273.05	92.09
GM-CV%	65.1	45.1	47.9	48.6	24.1
**$AUC_{0-\infty}$(h*ng/mL)**					
Mean±SD	87.40±72.37	139.36±74.03	130.97±77.85	301.06±119.08	102.42±28.35
GM	71.25	126.58	116.98	275.50	98.77
GM-CV%	65.9	45.7	49.0	49.3	28.9
**AUC_{0-24h}(h*ng/mL)**					
Mean±SD	70.38±39.58	119.60±38.59	106.04±31.00	239.14±68.49	94.80±21.22
GM	63.54	114.13	101.83	228.26	92.44
GM-CV%	45.8	33.0	30.8	34.7	24.2
**T_{max}(h)**					
Mean±SD	3.13±2.45	2.83±1.81	4.58±2.44	5.00±2.40	3.23±1.45
Median	2.25	2.50	4.00	4.50	3.50
Min ~ Max	1 ~ 8	1 ~ 8	2.5 ~ 12	2 ~ 8	1 ~ 6
**$t_{1/2z}$(h)**					
Mean±SD	5.79±3.56	5.81±2.56	6.07±3.10	7.81±3.91	5.39±1.73
GM	5.12	5.42	5.56	6.87	5.17
GM-CV%	50.5	38.7	43.2	58.6	29.7

Supplementary Table 2. Summary of main PK parameters of Ro18-5528 following single-dose dimdazenil administration.

Parameter	1.5 mg N=12	2.5 mg fasted N=12	2.5 mg fed N=12	5 mg N=12
**C_{max}(ng/mL)**				
Mean±SD	0.16±0.03	0.22±0.04	0.30±0.08	0.41±0.11
GM	0.16	0.21	0.29	0.40
GM-CV%	17.1	21.3	30.2	29.3
**$AUC_{0\text{-}t}$(h*ng/mL)**				
Mean±SD	7.66±1.65	10.66±2.01	15.45±4.31	20.66±5.70
GM	7.50	10.46	14.81	19.91
GM-CV%	21.5	21.0	32.6	29.9
**$AUC_{0\text{-}24h}$(h*ng/mL)**				
Mean±SD	1.26±0.43	2.10±0.67	3.50±1.17	4.18±1.21
GM	1.19	2.00	3.29	4.02
GM-CV%	37.5	32.9	40.7	30.3
**T_{max}(h)**				
Mean±SD	35.92±8.88	33.00±10.39	35.00±9.52	32.00±5.91
Median	35.92	30.00	36.00	36.00
Min ~ Max	23.83 ~ 48	24 ~ 48	24 ~ 48	24 ~ 36.02

Ro18-5528 cannot be fitted λ_z or $AUC_{\_\%Extrap}$ is greater than 20%, $AUC_{0\text{-}\infty}$、$t_{1/2z}$ are not included in the analysis.

Supplementary Table 3. Summary of main PK parameter of Ro46-1927 following 2.5 mg multiple-dose dimdazenil administration.

**Parameter**	2.5 mg/day (N=13)
**$C_{max,ss}$(ng/mL)**	
Mean±SD	9.64±1.93
GM	9.48
GM-CV%	19.0
**$AUC_{0-t,ss}$(h*ng/mL)**	
Mean±SD	117.09±31.98
GM	113.31
GM-CV%	27.0
**$AUC_{0-\infty,ss}$(h*ng/mL)**	
Mean±SD	117.64±32.05
GM	113.87
GM-CV%	26.9
**$AUC_{0-\tau}$(h*ng/mL)**	
Mean±SD	108.91±24.64
GM	106.36
GM-CV%	23.0
**$T_{max,ss}$(h)**	
Mean±SD	3.16±1.26
Median	3.00
Min ~ Max	1 ~ 6
**$t_{1/2z}$(h)**	
Mean±SD	5.33±1.25
GM	5.21
GM-CV%	22.6
**$R_{a(AUC)}$**	
Mean±SD	1.15±0.10
GM	1.15
GM-CV%	9.0
**$R_{a(Cmax)}$**	
Mean±SD	1.14±0.13
GM	1.13
GM-CV%	11.5

Supplementary Table 4. Summary of main PK parameter of Ro18-5528 following 2.5 mg multiple-dose dimdazenil administration.

Parameter	2.5 mg/day (N=13)
**$C_{max,ss}$(ng/mL)**	
Mean±SD	0.62±0.18
GM	0.60
GM-CV%	31.7
**$AUC_{0-t,ss}$(h*ng/mL)**	
Mean±SD	35.71±10.52
GM	34.14
GM-CV%	33.7
**$AUC_{0-\tau}$(h*ng/mL)**	
Mean±SD	14.22±4.17
GM	13.61
GM-CV%	32.8
**$T_{max,ss}$(h)**	
Mean±SD	5.27±4.23
Median	4.00
Min ~ Max	0 ~ 12
**$R_{a(AUC)}$**	
Mean±SD	6.05±0.79
GM	6.00
GM-CV%	12.9
**$R_{a(Cmax)}$**	
Mean±SD	3.10±0.38
GM	3.08
GM-CV%	12.6

1. The last administration of Ro18-5528 in the 2.5mg multiple group could not be fitted λ_z or $AUC_{\_\%Extra.}$ Extrap is greater than 20%, so $AUC_{0-\infty,ss}$ and $t_{1/2z}$ are not included in the analysis.

2. AUC_{0-24h} could not be fitted for all subjects, so AUC_{0-t} was alternatively used to calculate Ra(AUC).

Supplementary Table 5. Dimdazenil exposure parameters after a single oral administration of dimdazenil in men and women

Parameter	Men<br>N=35	Women<br>N=14
**C_{max}(ng/mL/mg)**		
Mean±SD	13.54±3.19	15.73±4.67
GM	13.18	15.18
GM-CV%	24.2	27.5
**AUC_{0-t}(h*ng/mL/mg)**		
Mean±SD	85.05±30.85	89.41±52.39
GM	79.79	79.43
GM-CV%	37.9	50.4
**$AUC_{0-\infty}$(h*ng/mL/mg)**		
Mean±SD	86.01±31.04	90.25±52.30
GM	80.73	80.48
GM-CV%	37.8	49.3
**AUC_{0-24h}(h*ng/mL/mg)**		
Mean±SD	83.24±27.80	87.86±47.53
GM	78.72	79.26
GM-CV%	35.4	46.9

Supplementary Table 6. Ro46-1927 exposure parameters after a single oral administration of dimdazenil in men and women

Parameter	Men<br>N=35	Women<br>N=14
**C_{max}(ng/mL/mg)**		
Mean±SD	3.63±0.64	4.59±0.80
GM	3.57	4.54
GM-CV%	17.6	16.1
**AUC_{0-t}(h*ng/mL/mg)**		
Mean±SD	51.26±25.10	54.34±41.39
GM	46.53	46.23
GM-CV%	45.6	56.5
**$AUC_{0-\infty}$(h*ng/mL/mg)**		
Mean±SD	52.89±25.92	55.16±42.11
GM	47.95	46.90
GM-CV%	46.1	56.6
**AUC_{0-24h}(h*ng/mL/mg)**		
Mean±SD	44.11±13.67	47.15±24.25
GM	42.06	43.20
GM-CV%	32.5	42.1

Supplementary Table 7. Ro18-5528 exposure parameters after a single oral administration of dimdazenil in men and women

Parameter	Men N=26	Women N=10
**C_{max}(ng/mL/mg)**		
Mean±SD	0.09±0.02	0.10±0.02
GM	0.09	0.10
GM-CV%	27.8	17.8
**AUC_{0-t}(h*ng/mL/mg)**		
Mean±SD	4.40±1.14	4.77±0.95
GM	4.25	4.69
GM-CV%	27.6	19.9
**AUC_{0-24h}(h*ng/mL/mg)**		
Mean±SD	0.77±0.24	1.02±0.24
GM	0.74	0.98
GM-CV%	30.8	27.9

Supplementary Table 8. The duration of time to plasma concentration reaches 20%C_{max} after drug administration.

Dose	Phase	Mean	SD	CV percent
2.5 mg single dose	T_{onset}	0.25	0.11	42.32
	T_{offset}	9.56	3.23	33.78
	Duration of time	9.31	3.30	35.50
2.5 mg multiple dose Day 1	T_{onset}	0.53	0.32	60.06
	T_{offset}	9.41	2.87	30.54
	Duration of time	8.88	2.72	30.66
2.5 mg multiple dose Day 5	T_{onset}	0.46	0.29	63.73
	T_{offset}	9.91	2.52	25.39
	Duration of time	9.45	2.33	24.69

T_{onset}: the time when the plasma concentration reaches 20% C_{max} after drug administration;
T_{offset}: the time to eliminate the plasma concentration to 20% C_{max};
Duration of time: the time for the plasma concentration to be maintained at or above 20%C_{max};
